# International Comparison of Self-Concept, Self-Perception and Lifestyle in Adolescents: A Systematic Review

**DOI:** 10.3389/ijph.2022.1604954

**Published:** 2022-09-29

**Authors:** Natacha Palenzuela-Luis, Gonzalo Duarte-Clíments, Juan Gómez-Salgado, José Ángel Rodríguez-Gómez, Maria Begoña Sánchez-Gómez

**Affiliations:** ^1^ Canary Islands University Hospital, Santa Cruz de Tenerife, Spain; ^2^ Cátedra de Enfermería, University of La Laguna, San Cristobal de La Laguna, Spain; ^3^ Cieza Este Health Centre, Servicio Murciano de Salud, Murcia, Spain; ^4^ Department of Sociology, Social Work, and Public Health, Faculty of Labour Sciences, University of Huelva, Huelva, Spain; ^5^ Escuela de Posgrado, Universidad de Especialidades Espíritu Santo, Guayaquil, Ecuador; ^6^ Department of Nursing, Faculty of Health Sciences, University of La Laguna, San Cristóbal de La Laguna, Spain

**Keywords:** exercise, questionnaire, adolescent, lifestyle, self-concept, self-perception

## Abstract

**Objectives:** Adolescence is considered a vital time to address healthy attitudes and values towards an effective transition to adulthood. The aim of this review was to analyse self-concept, self-perception, physical exercise, and lifestyle in the late adolescent population.

**Methods:** Systematic review of studies assessing the results by the Rosenberg Self-Esteem Scale, the General Health Questionnaire, the Activity Questionnaire for Adolescents, and the Health Behaviour in School-aged Children questionnaires in late adolescents. The PRISMA recommendations were followed. The CASPe quality-check system was applied, excluding articles with a score <8.

**Results:** 1589 studies were found, and 69 articles were selected. Adolescents with high self-concept and self-perception tend to be emotionally stable, sociable, and responsible. No significant differences were found regarding self-concept and self-perception between different countries, but there were differences between men and women. Physical activity and healthy diet improve self-concept and perception of body image.

**Conclusion:** Self-concept and self-perception are associated with responsibility, stability, and mental strength. Most healthy behaviours during adolescence are followed during adulthood. Socio-cultural level of Health Science students is a differential factor for overweight and obesity.

## Introduction

Satisfaction with one’s life is related to a lower number of illnesses, increased happiness, and better emotional well-being [1], and the adolescence is considered a key time in life to address these emotional, social, and physiological aspects that affect their development and well-being [2].

Many authors coincide that adolescence begins at puberty and ends with the complete development of the organism and the onset of adulthood [2–5]. During this transition, there is a continuous process of self-assertion in the pursuit of independence where individual and social identity are consolidated [5]. The World Health Organisation (WHO) considers late adolescence to be the period between 19 and 24 years of age. By this time, the person is preparing for their profession and adult life, so it is determinant to promote healthy lifestyle habits, which in most cases are maintained throughout adulthood [5–7]. In Hispanic cultures, there is also a characteristic family model with dependence on care, security, protection, and social policies of the welfare state [8]. In any case, in order for the subject to adopt and develop the competences, skills, and values that will enable them to make the transition to adulthood effectively, it is necessary to focus on the positive development of the adolescent [9].

Self-concept, self-esteem, and self-perception are interrelated and condition the lifestyle and health-related habits of individuals, particularly of adolescents.

Self-concept is understood as the set of feelings that the subject has about him/herself. In adolescents, it is important since it is an indicator of an adequate physical, cognitive, behavioural, affective, and social integration of the individual [10]. It is related to the individual own interpretation of the world [5], and it is also influenced by values, cultural expectations, and personal relationships [11]. The higher the self-concept, the greater the feeling of satisfaction [2, 12]. Following this line of argument, self-esteem is known as the sense of personal efficacy or self-efficacy, the assurance of self-worth, and the right to live and be happy [11, 13, 14]. Self-esteem is related to self-respect and self-acceptance [15]. It is a protective factor against unhealthy behaviours such as anxiety, depression, and suicidal ideation [13, 16].

Finally, self-perception is important for understanding how individuals think, behave, and relate to others. It is understood that self-perception includes those internally conscious and organised concepts that the individual has about him/herself. It is related to a greater or lesser extent to age, low levels of schooling, income, race, marital status, smoking, physical activity, alcohol consumption, presence of chronic morbidity, and body mass index [17, 18].

Overweight and obesity are among the most important problems in adolescence due to their psychological and social impact. The expansion of new technologies has influenced the prevalence of sedentary lifestyles, unbalanced and hypercaloric diets, and social changes [19]. Moreover, in terms of morbidity, there has been an increase of the risk factors for cardiovascular disease and other chronic pathologies in adulthood [5, 20]. In Spain, the prevalence of overweight and obesity in this age group is estimated at 15.5% in women and 16.5% in men.

Health education approaches are decisive in adolescence [20]. Thus, the practice of physical exercise is key to prevent overweight and obesity. It is responsible for the proper functioning of the body and is part of physical well-being and a healthy lifestyle [21–23]. It is also related to increased cognitive competence [21, 22] and prevents chronic diseases such as obesity, cardiovascular diseases, and metabolic syndrome [23]. According to various studies, men devote more time to physical activity [21, 22].

Living a healthy lifestyle has an impact on the prevention of cardiovascular diseases, which are the main cause of premature death in industrialised countries. The influence of lifestyle is also determinant to reduce the onset of pathologies such as type 2 diabetes mellitus, hypertension, dyslipidaemia, overweight, and obesity [24, 25]. Otherwise, the psychological state that most influences lifestyle is stress, which is associated with poorer health by increasing the risk of heart disease, cancer, and/or suppression of the autoimmune system [25–27]. Increased job dissatisfaction, work intensity, inflexible working hours, or difficulty in work-life balance are associated with increased sick leave due to stress, anxiety, or depression [25–28].

Comparing the self-concept, self-esteem, self-perception, and physical exercise that young people have depending on their culture is fundamental to understand the actions for improvement that should be carried out on lifestyle. Knowledge on whether there are differences with respect to the environment and socio-cultural level is necessary to understand how it influences the individual. It also would facilitate the positive approach and development of the adolescent. In order to assess the self-concept, self-esteem, self-perception, physical activity, and lifestyle of adolescents, this research team previously conducted a systematic review titled “Questionnaires assessing adolescents’ self-concept, self-perception, physical activity, and lifestyle: a systematic review” [26]. The aim was to determine which questionnaires are optimal for the assessment of these concepts. In conclusion, the Rosenberg Self-Esteem Scale (RSES), the General Health Questionnaire 12 (GHQ-12), the Physical Activity Questionnaire for Adolescents (PAQ-A), and the Health Behaviour in School-aged Children (HBSC) are valid and reliable tools for the assessment of self-concept, self-esteem, self-perception, physical activity, and lifestyle, respectively.

### Objectives

1. To analyse the results provided by the following questionnaires: Rosenberg Self-Esteem Scale (RSES), General Health Questionnaire 12 (GHQ-12), Physical Activity Questionnaire for Adolescents (PAQ-A), and Health Behaviour in School-aged Children (HBSC), on the late adolescent population.
2. To evaluate possible differences in self-concept, self-esteem, self-perception, physical exercise, and lifestyle of young people depending on their culture.

## Methods

**FIGURE 1:**
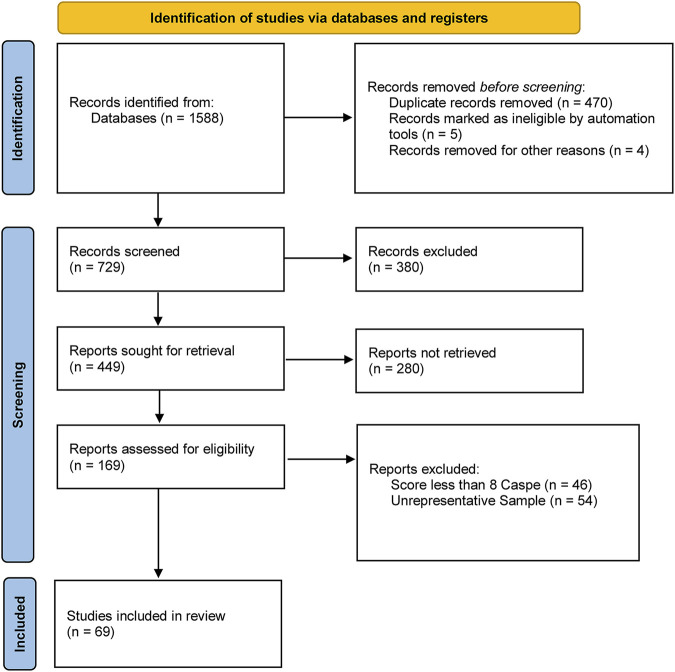
PRISMA flow chart (Spain, 2022).

A systematic review of the Scoping Review type was developed, following the recommendations of the Preferred Reporting Items for Systematic reviews and Meta-Analyses, also known as the PRISMA statement [29] (Figure 1). A search was carried out in different databases on self-concept, self-esteem, self-perception, physical exercise, and lifestyle of late adolescents at an international level.

### Eligibility Criteria

There is no prior record of the study protocol. However, any study was acceptable regardless of its design as long as it assessed self-concept, self-esteem, self-perception, physical activity, and lifestyle with the RSES, GHQ-12, PAQ-A, and HBSC instruments on late adolescents. Therefore, the study population was late adolescents. Articles in English, Portuguese, and Spanish were included. The exclusion criteria were: studies not exceeding a score of 8 in the Critical Appraisal Skills Programme Español (CASPe) [30]; publications without a scientific basis, with an unrepresentative sample (children and/or adults), or whose representativeness was not stated when describing the sample; articles whose statistical significance was not stated or whose results were not statistically significant; questionnaires with Cronbach’s alpha lower than 0.75.

### Sources of Information

All articles meeting the eligibility criteria were selected. The PICO method was used for the investigation question, considering as patient/population: teenagers and undergraduates; Intervention: questionnaires about lifestyle; Comparator: International late adolescents; Outcome: self-identity, introspection, workout, and way of living. The date of the last search is 07 March 2022.

### Search Strategy

DECS and MeSH descriptors and Boolean operators were used (Table 1). A preliminary search was conducted in the following databases: Biblioteca Virtual de la Salud (BVS) (Health Virtual Library), Pubmed, Cuiden, Dialnet, Cochrane, Scielo, and Ministerio de Sanidad, Consumo y Bienestar Social de España (Table 1). Information was expanded by reference searching where appropriate. The search limits were set for full text availability, publication in the last five years, and English, Spanish, and/or Portuguese language.

**TABLE 1:** Search strategy (Spain, 2022).

Date of search	Type of search	Database	DECS-MeSH combination	Selection/Results
6/11/19	Electronic	Biblioteca Virtual de la Salud (BVS)	Ejercicio AND autopercepción	5/783
06/11/19	Electronic	Biblioteca Virtual de la Salud (BVS)	Estilo de vida AND autopercepción	5/94
13/12/19	Electronic	Cochrane	Adolescente AND estilo de vida	4/10
13/12/19	Electronic	Cochrane	Adolescente AND autoestima	3/9
20/12/19	Electronic	Cochrane	Adolescente AND ejercicio	5/31
02/01/20	Electronic	Cuiden	Autopercepción AND cuestionario	2/72
02/01/20	Electronic	Cuiden	Autoconcepto AND cuestionario	3/21
10/01/20	Electronic	Cuiden	Actividad física AND adolescentes AND cuestionario	5/31
01/07/20	Electronic	Scielo	Ejercicio AND autoestima	5/34
30/12/20	Electronic	Dialnet	Self-concept AND questionnaire	4/28
01/07/20	Electronic	Pubmed	Self-perception AND questionnaire	5/47
13/01/21	Electronic	Pubmed	Exercise AND self-concept AND questionnaire	5/23
13/01/21	Electronic	Pubmed	Self-concept AND questionnaire AND teenager	5/53
10/01/20	Electronic	Medline	Exercise AND self-perception AND teenager	4/32
02/01/20	Electronic	Medline	Self-concept AND questionnaire AND lifestyle	6/100
29/10/21	Manual	Ministerio de Sanidad, Consumo y Bienestar social	Estilo de vida AND cuestionario	2/200

Source: self-elaborated.

Search limits: open access articles with full text available, articles published in the last 5 years, studies in Spanish, English, and Portuguese.

### Study Selection Process

A critical reading of the articles was carried out and they were assessed using the CASPe [30] system. The CASPe classification was determined by the type of research of each article, where the first two questions were elimination questions. The accepted level for the critical reading was 8 points irrespective of the type of study (Supplementary File S1). Quality assessment was carried out independently by peers. Divergences were resolved by debate and consensus of the investigators. The Grading of Recommendations, Assessment, Development and Evaluation (GRADE) [31] system was used to assess the level of recommendation. High and moderate-quality articles were selected (Supplementary File S1). To assess the clinimetric quality of the instruments, COnsensus-based Standards for the selection of health status Measurement INstruments (COSMIN) [32] tool was applied (Table 2). Automation tools were not used.

**TABLE 2:** Assessment of the COSMIN instruments (Spain, 2022).

	Self-concept	Self-perception	Physical exercise	Lifestyle
	RSES^a^	GHQ-12^b^	PAQ-A^c^	HBSC^d^
1. Formulate the objective of the review.	Yes	Yes	Yes	Yes
2. Formulate the eligibility criteria.	Yes	Yes	Yes	Yes
3. Conduct a bibliographic search.	Yes	Yes	Yes	Yes
4. Select abstracts and full-text articles.	Yes	Yes	Yes	Yes
5. Extract data on the characteristics of the measuring instruments included and information on feasibility and interpretability.	Yes	Yes	Yes	Yes
6. Assess content validity.	Yes	Yes	Yes	Yes
7. Assess internal structure.	Yes	Yes	Yes	Yes
8. Assess reliability and measurement error	Yes	Yes	Yes	Yes
9. Assess other measurement properties (criterion validity, hypothesis testing for construct validity, and sensitivity).	Yes	Yes	Yes	Yes
10. Formulate recommendations.	Yes	Yes	Yes	Yes
11. Select a systematic review.	Yes	Yes	Yes	Yes
Internal consistency (Cronbach’s alpha)	0.85–0.88	0.76	0.77–0.84	0.90

Source: self-elaborated.

a RSES: Rosenberg self-esteem scale.

b GHQ-12: general health questionnaire-12.

c PAQ-A: physical activity questionnaire for adolescents.

d HBSC: health behaviour in school-aged children.

### Data Extraction Process

This was performed qualitatively by the researchers. Discrepancies were identified and extraction was agreed upon to produce the data list.

### Data List

To structure the data, all results compatible with the use of the RSES, GHQ-12, PAQ-A, and HBSC questionnaires on late adolescents were searched at international level. The results of the different items were compared on the basis of self-concept, self-esteem, self-perception, physical exercise, and lifestyle among young people with socio-cultural differences. Table 1 expresses the results consistent with each domain.

### Assessment of the Risk of Bias in the Individual Studies

During the critical reading of the articles, their internal structure, validity, and reliability were observed. The assessment of the degree of recommendation, as well as the analysis of the study population, the variables, the interventions used, and the results are described in Supplementary File S1.

### Measures of Effect

The following questionnaires were used: RSES to assess self-concept; the GHQ-12 for the assessment of self-perception; the PAQ-A to measure physical exercise; and the HBSC study, where the lifestyle of adolescents is reflected. Table 2 describes the clinimetric quality of these instruments using the COSMIN tool [32].

### Synthesis Methods

All articles with a moderate and high level were gathered using the GRADE system [31]. All articles using the RSES, the GHQ-12, the PAQ-A, and the HBSC on adolescents at the international level were analysed and selected according to the COSMIN protocols [32] (Table 2).

### Assessment of Publication Bias and Certainty of Evidence

A critical reading of the articles selected from the consulted databases was carried out. This systematic review was peer-reviewed by the researchers, deciding by consensus those issues that were not in agreement. The CASPe [30] system was applied to the articles used for the development of this systematic review, and this work was also assessed once completed to self-check for possible methodological biases, receiving a score of 10/10 points. The recommendations of the PRISMA [29] statement were followed as a self-checklist (Supplementary File S2).

## Results

### Study Selection

After the search, 1589 studies were identified. The total number of articles eliminated for being duplicates, non-retrievable publications, not meeting the study objectives, and/or not exceeding the CASPe [30] score with more than 8 was 1519. A total of 69 articles were selected as represented in the flow chart (Figure 1). The 4 methodological articles PRISMA [29], CASPe [30], GRADE [31], and COSMIN [32] were also added.

### Study Characteristics

These are described in Supplementary File S1. 61 case-control studies, 4 systematic reviews, 1 clinical trial, 2 cohort studies, and 1 clinical prediction rule study were selected.

### Risk of Bias of Individual Studies

This was assessed with the CASPe [30] critical appraisal tool (Supplementary File S1).

### Results of Individual Studies

#### Self-Esteem and Self-Concept

The Rosenberg Self-Esteem Scale (RSES) measures the overall self-esteem of the individual. It is a two-dimensional scale: positive self-esteem (self-confidence or personal satisfaction) and negative self-esteem (self-contempt or personal devaluation) [14, 15]. It is based on 10 items with a 4-point Likert scale. Five items are scored positively and five items, negatively. The total score ranges from 10 to 40, with 10 being the lowest self-esteem score and 40 the highest [15, 33–43].

As already mentioned, self-concept and self-esteem are linked. Thus, according to the study by Daniela-Calero A, et al. [10], having high self-esteem and self-concept in adolescence is significantly related to being emotionally stable, and a social and responsible subject.

Gálvez-Casas A, et al. [9] showed that males show a prevalence of overweight and females of obesity. The highest scores for general self-concept were for people whose weight was within normal limits. For this reason, the authors highlight the need to contribute to the improvement of physical self-concept as it favours the balanced development of the adolescent’s personality [9]. The research by Molero D, et al. [41] states that physical self-concept is related to the age of the subject. Scores on this concept improve over the years, being higher at the beginning and intermediate stages of life, and lower during adolescence. In the scales of physical ability and physical attractiveness, it was the adults who obtained better scores. This data could indicate that there is better physical acceptance at older ages.

#### Self-Perception

The General Health Questionnaire (GHQ) is a tool for measuring minor psychiatric morbidity in community, primary care, or medical-surgical outpatient settings. It assesses a person’s self-perceived health over the past six months [44]. The GHQ 12-item questionnaire is the most widely used tool for the assessment of non-psychotic psychiatric disorders [45–67], and it measures mental health in specific groups such as adolescents [45–51]. It has been adapted to and translated into 38 languages and applied in national health surveys and studies carried out by the WHO [51]. It has a two-dimensional structure: one part assesses depression and the other, social dysfunction [45, 51].

A study in a hospital institution in the city of Medellín with the GHQ-12 [46] surveyed 29476 people over 16 years of age and found out that the perception of psychological well-being or distress of both the self and social functioning are related to intrinsic factors (motivational, cognitive, emotional, and personality traits) [46]. In the study by Brabete AC, et al. [49], where the surveyed population is from Romania, it is women who have a higher prevalence of mental illness. During the adolescence period, a greater differentiation in this aspect between males and females begins to emerge.

Videra-García A, et al. [3] indicate a significant sex differentiation, and show that boys have a better assessment of their health. The study highlights the fact that females score higher on health perception, although this is not true since it is females who have a higher prevalence of mental illness. Thus, this result indicates greater health problems in girls than in boys. This study concludes that males have a better physical self-concept and self-perception of health than females, with a confidence level of 95% [3].

Inchley J, et al. [66] state that Spanish adolescents’ perceived support from their families is high, but scores on the ease of communication with parents decrease. However, indicators of emotional well-being receive positive results.

Soria-Trujano R, et al. [67] concluded that it is Mexican women who present more cases of depression. Also, health science students indicate a greater presence of anxiety due to work experience in health centres, which leads to sleep problems [67].

#### Lifestyle

The Health Behaviour in School-aged Children (HBSC) measures young people’s lifestyle habits and creates health promotion tools. It evaluates socio-demographic variables, diet, hours of sleep, risky consumption, oral hygiene, sexual behaviour, physical activity and sedentary behaviours, family context, leisure time, schooling, social environment, general health and psychological status, and socio-economic data. It is an initiative promoted by the WHO at the international level. Its latest version was published in 2020.

The report “*Spotlight on adolescent health and well-being*” [68] establishes a comparison of the lifestyle of young Spaniards and their peers in other European countries. The main findings are as follows: the level and quantity of physical exercise recommended by the WHO has worsened: less than 1 in 5 adolescents do so. Spain became involved in this research in 1986 [69, 70].

Regarding nutritional habits, the majority of young people at international level do not follow the indications, which is detrimental to their healthy development. For this reason, the level of overweight and obesity has increased considerably, affecting 1 in 5 adolescents [7].

The results of the study “Healthy lifestyles in Nursing students of the Cooperative University of Colombia” [25] show that 27.3% of Nursing students are overweight and 7.8% are morbidly obese. The students indicate that they prefer to do physical exercise outside the university, but the lack of practice is striking [25].

Despite the cultural level of university students in Health Sciences and their extensive knowledge of healthy nutrition [19], they do not act accordingly. It is considered essential to promote health education as a promotional tool for young people and to generate changes for their own benefit and for the benefit of society [19].

Salas-Salvadó J, et al. [23] report that people who follow a Mediterranean diet have a lower chance of suffering from cardiovascular diseases. A follow-up of almost 5 years was carried out, where the results indicate that the probability of suffering a primary cardiovascular event was 30% lower in people following a traditional diet. Also, the consumption of nuts and olive oil is associated with a 40% lower risk of diabetic retinopathy and a higher probability of reversing the metabolic syndrome [23]. On the other hand, although substance use has decreased, the number of adolescents who use alcohol and tobacco remains high. 1 in 5 adolescents have been drunk two or more times in their lifetime and 1 in 6 respondents have smoked in the last 30 days. Regarding risky sexual behaviour, 1 in 4 adolescents have had unprotected sex [68].

Soria-Trujano R, et al. [67] compare Mexican health science professionals and show that, among nurses, there is a higher number of consumers of toxic substances such as alcohol and tobacco. It is men who stand out in their consumption, and women have a significant intake. This contrasts with the knowledge they have about the health problems associated with the consumption of these substances. Although it can reduce the level of stress, it is detrimental to academic performance [67].

In the research carried out by Hernando A, et al. [6] boys have a worse academic performance than girls, but their time among friends is greater. Women show a decrease in physical exercise and a reduction in the number of hours of sleep [6].

#### Physical Exercise

The PAQ-A (Physical Activity Questionnaire for Adolescents) assesses the physical exercise of the person in the last 7 days. It consists of 9 questions that measure aspects of the physical exercise performed by the adolescent. It also provides information on whether the person has been ill. It is evaluated by means of a scale of 1 to 5 points that establishes a graduation of the level of physical activity carried out. It allows us to know at what time of the week the person is most active [22, 68, 69].

According to the study by Rizo Baeza MM, et al. [5], overweight and obese young people spend more hours doing physical activity than those of normal weight, followed by those of low weight, who dedicate the least hours to their physical activity [5], the difference being statistically significant.

According to Martínez-Gómez D, et al. [22], physical activity measured by the PAQ-A questionnaire presents indicators of adiposity, bone mineral content, psychological indicators, and heart rate variability [22].

Ruiz-Ariza A, et al. showed that men are more attracted to physical activity than women [20].

The level of physical activity in a sample of adolescents aged between 14 and 17 years was analysed in Colombia [70]. Statistically significant differences were found between high and low levels of physical exercise according to the PAQ-A questionnaire. Young people with higher levels of physical activity showed an increased interest in physical exercise. It is noteworthy that females would complete workouts twice a week for four years but that this activity did not increase with age. They conclude by commenting that students with better results in the PAQ-A are more likely to obtain better physical conditions (strength, endurance, and speed).

According to Rincón Herrera AD, et al. [71], Colombian adolescents do not increase their level of physical exercise. This is true, according to Ruiz Ariza A, et al. [20], in Spanish male adolescents, who are more attracted to sports than their female counterparts. However, in the study conducted by Rizo Baeza MM, et al. [5], there is controversy with respect to Rincón Herrera AD, et al. [71] since, according to him, young people with weight problems practice more sport than the rest. However, the opposite is true for Colombian adolescents.

Synthesis results: the results of the synthesis of the individual works are shown in Supplementary File S1. The assessment of the questionnaires using the COSMIN [32] tool is shown in Table 2. Table 3 summarises the results of this systematic review.

**TABLE 3:** Results synthesis (Spain, 2022).

Concept	National results	International results
Self-concept and self-esteem	No differences reported	
Self-perception	No cultural differences are found with regard to the prevalence of mental illnesses such as depression or anxiety.	
Physical exercise	Men are more interested in sports than women.	Young Colombian women do not increase their level of physical exercise.
	Young Spaniards with weight problems do more sport than the rest.	Normal-weight young Colombian men and women do more sport.
Lifestyle	Health science students do not act on the knowledge they have learnt about healthy lifestyles.	
	There are more Nursing students who are overweight, obese, and substance abusers.	

Publication bias: all articles that did not obtain a score greater than or equal to 8 in the CASPe [30] tool were eliminated, thus avoiding bias in the results of this systematic review.

## Discussion

Self-assessment of health should be incorporated into health surveys on a regular basis, as self-perception of health is a good predictor of morbidity and mortality [31]. Men tend to suffer from life-threatening health problems. In contrast, women suffer to a greater extent from disabling conditions due to chronic diseases. For this reason, there is an increase in women’s negative self-perception [72]. A person’s lifestyle contributes to the development of chronic non-communicable diseases, which are the main cause of morbidity and mortality [24]. The resulting public health problem justifies the search for instruments to assess the state of self-concept, self-perception, physical exercise, and lifestyle. Valid and reliable assessment tools are required [26].

As results with a moderate-high degree of recommendation (GRADE) [31], it should be noted that the fact that adolescents have a high self-concept predisposes them to be emotionally stable, sociable, and responsible [10]. Some authors attach great importance to the improvement of physical self-concept. The reason for this is the balanced development of the young person’s personality. Being a person with a normal body mass index predisposes him or her to a better general self-concept. However, the number of overweight and obese people continues to increase [9]. Physical acceptance improves as we get older. Adults score higher on attractiveness and physical ability. This is an indicator of the relationship between age and self-concept. In several articles, this fact is linked to the social pressure that exists among the youth [41]. No differences are reported between adolescents from different countries or socio-cultural backgrounds. All authors reach the same conclusions: self-concept is fundamental in the adolescent stage; it is linked to self-esteem and directly influences the psychological and physical state of the person. They suggest that, in adulthood, self-concept and self-esteem are better scored. However, improving self-concept from an early age is key, as it favours the balanced development of the adolescent's personality [9, 10, 33–40].

With a moderate level of recommendation, it was found that men have a better physical self-concept and self-perception of health than women [2]. The prevalence of mental illnesses such as depression and anxiety is higher among females [72]. In turn, Health Science students have a higher prevalence of anxiety, that leads to sleep deprivation [49, 67]. Spanish adolescents rate the support from their relatives as high, but this rating decreases when it comes to being able to communicate with their parents. It is worth noting that emotional well-being receives positive results [68]. In conclusion, no cultural differences are found with regard to the prevalence of mental illnesses such as depression or anxiety in women. This fact should be highlighted since an improvement would be expected in countries with an educational and social system such as Spain. From an early age, there is an emphasis on health education and a multitude of tools are provided for the positive development of adolescents. However, it is boys who have a better assessment of their health [3, 42, 43, 48, 49, 68, 71].

Health science students have knowledge and tools to improve their lifestyle; however, they do not apply this knowledge to their own benefit [5, 18, 24, 72]. Following a correct diet prevents the onset of chronic non-communicable diseases [23]. The Nursing degree has a higher number of consumers of toxic substances such as alcohol and tobacco. Men stand out despite the fact that women have a high intake. It is striking that they are not capable of putting their knowledge into practice, given that they will be responsible for communicating a message of prevention and health promotion to the general population [67, 71, 72].

Regarding cultural differences, the data provided by the RSES on self-esteem and self-concept do not report significant differences between adolescents from different countries. This concept is fundamental during the youth stage because it directly influences the psychological and physical state of the person [9–33]. Something similar occurs with the GHQ-12. Self-perception of adolescents is fundamental to avoid psychological illnesses such as depression, anxiety, or suicidal thoughts. It is necessary to focus on the positive development of adolescents and, especially, on this concept in women, since it is they who have the highest prevalence of psychiatric pathology at an international level [3, 42, 43, 48, 49, 67, 68, 73].

Physical exercise, assessed by the PAQ-A questionnaire, is shown to be lower in both Colombian and Spanish women than in men [20, 70]. However, Spanish adolescents with overweight and obesity problems do more sports than young Colombians with the same health problem [5, 70]. Body weight has an impact on the hours of physical exercise adolescents take, although, it is men who are more attracted to sports, devoting more hours to them [20, 22]. Nevertheless, fewer than 1 in 5 adolescents engage in physical exercise [6]. It would be interesting to delve on the reasons for the decrease in attraction to physical activity among women, and the question arises as to whether body image influences interest in undertaking a higher level of physical exercise.

In conclusion, comparing the self-concept, self-esteem, self-perception, and physical exercise of young people depending on their culture is essential to understand the actions to be taken to improve lifestyles. Knowing whether there are differences with respect to the environment and socio-cultural level is key to understand whether it influences the individual. Understanding and promoting a healthy lifestyle in adolescents helps to target and implement interventions from a preventive point of view. It also facilitates positive adolescent approach and development, and it reduces economic costs, since the correct approach to young people avoids future complications such as chronic non-communicable diseases (depression, anxiety, overweight, obesity, among others) and hospital admissions for this reason.

A healthy young and adult population is beneficial not only for the individual but also for their environment, which benefits from having a productive and healthy community. Improving self-concept increases the likelihood of being a more sociable, responsible, and emotionally stable person, which is linked to a greater self-esteem.

### Limitations

This study is conditioned by the validity and reliability of the data provided in the selected scientific articles. It is also conditioned by the search for articles in English, Portuguese, and Spanish. It is influenced by the selection processes of studies where the CASPe [30] and the GRADE system are taken into account [31]. The aim is to review the literature and describe the self-concept/self-esteem, self-perception, physical activity, and lifestyle of adolescents at an international level. The results are extracted from the questionnaires selected for the assessment of the four variables mentioned above. This systematic review would provide more representative data if further research were to be carried out using the same methodology and broadening the selection of articles.

## Abbreviations

BVS, biblioteca virtual de la salud (Health Virtual Library); CASPe, critical appraisal skills programme español; COSMIN, consensus-based standards for the selection of health measurement instruments; DECS, Descriptores en Ciencias de la Salud; GHQ-12, general health questionnaire-12; GRADE, grading of recommendations, assessment, development and evaluation; HBSC, health behaviour in school-aged children; MeSH, medical subject headings; PAQ-A, physical activity questionnaire for adolescents; PICO, patient/population; intervention; comparator; outcome; PRISMA, preferred reporting items for systematic reviews and meta-analyses; RSES, Rosenberg self-esteem scale; WHO, world health organisation.
