# Supplementary material for: International Comparison of Self-Concept, Self-Perception and Lifestyle in Adolescents: A Systematic Review
Source: Int J Public Health. 2022 Sep 29;67:1604954. doi: 10.3389/ijph.2022.1604954 (PMC9556634; doi:10.3389/ijph.2022.1604954)
Supplement: Supplementary file 2 [file Table2.docx]

***Supplementary File 2.*** *PRISMA self-assessment.*

| **Section** | **Subject** | **Item in verification list** | **Localisation of item in publication (Page)** |
| --- | --- | --- | --- |
| **Abstract** | **Title** | Identify the report or publication as a systematic review. | 2-3 |
|  | **Background** |  | 2-3 |
|  | Objectives | Provide an explicit statement of the main objectives or questions addressed by the review. | 2-3 |
|  | **Methods** |  | 2-3 |
|  | Eligibility criteria | Specify the inclusion and exclusion criteria for the review. | 2-3 |
|  | Data sources | Specify the data sources (e.g. databases, records) used to identify the studies and the date of the last search that was made in each of these sources. | 2-3 |
|  | Risk of bias of individual studies | Specify the methods used to assess the risk of bias of the individual studies included. | 2-3 |
|  | Results synthesis | Specify the methods used to present and synthesise the results. | 2-3 |
|  | **Results** |  | 2-3 |
|  | Included studies | Provide the total number of included studies and participants and summarise the relevant characteristics of the studies. | 2-3 |
|  | Results synthesis | Present the results of the main outcomes and preferably indicate the number of included studies and participants in each study. If a meta-analysis has been performed, indicate the abstract estimator and the confidence or credible interval. If comparing groups, describe the effect direction (e.g. which group was favoured). | 2-3 |
|  | **Discussion** |  | 2-3 |
|  | Evidence limitations | Provide a brief summary of the limitations of the evidence included in the review (e.g., risk of bias, inconsistency -heterogeneity-, and imprecision). | 2-3 |
|  | Interpretation | Provide a general interpretation of the results and their important implications. | 2-3 |
|  | Other |  | 2-3 |
|  | Funding | Specify the main source of funding for the review. | 2-3 |
|  | Registration | Provide the name and registration number. | 2-3 |
| **Introduction** | Justification | Describe the justification for the review in the context of existing knowledge. | 4-6 |
|  | Objectives | Provide an explicit statement of the objectives or questions addressed by the review. | 6 |
| **Methods** | Eligibility criteria | Specify the inclusion and exclusion criteria for the review and how the studies were grouped for synthesis. | 7 |
|  | Data sources | Specify all databases, records, websites, organisations, reference lists, and other search or consult resources to identify the studies. Specify the date each resource was last searched or consulted. | 7 |
|  | Search strategy | Present the full search strategies for all databases, records, and websites, including any filters and limits used. | 7 |
|  | Study selection process | Specify the methods used to decide whether a study meets the review's inclusion criteria, including how many review authors screened each record and each retrieved publication, whether they worked independently and, if applicable, details of any automation tools used in the process. | 7 |
|  | Data extraction process | Indicate the methods used to extract data from reports or publications, including how many reviewers collected data from each publication, whether they worked independently, the processes for obtaining or confirming data on behalf of the study researchers, and, if applicable, details of the automation tools used in the process. | 8 |
|  | Data list | List and define all outcomes for which data were sought. Specify whether all results compatible with each outcome domain were sought (e.g. for all measurement scales, time points, analyses) and, if not, the methods used to decide which results were to be collected. | 8 |
|  |  | List and define all other variables for which data were sought (e.g., participant and intervention characteristics, funding sources). Describe all assumptions made about any missing or uncertain information. | 8 |
|  | Assessment of risk of bias of individual studies | Specify the methods used to assess the risk of bias of the included studies, including details of the tools used, how many review authors assessed each study and whether they worked independently and, if applicable, details of the automation tools used in the process. | 8 |
|  | Effect measures | Specify, for each outcome, the measures of effect (e.g. risk ratio, mean difference) used in the synthesis or presentation of the results. | 8 |
|  | Synthesis methods | Describe the process used to decide which studies were eligible for each synthesis (e.g. by tabulating the characteristics of the intervention studies and comparing them with the intended groups for each synthesis). | 8 |
|  |  | Describe any methods required to prepare data for presentation or synthesis, such as the handling of missing data in abstract statistics or data conversions. | 8 |
|  |  | Describe the methods used to tabulate or visually present the results of individual studies and their synthesis. | 8 |
|  |  | Describe the methods used to synthesise the results and justify your choices. If a meta-analysis has been performed, describe the models, the methods used to identify the presence and extent of statistical heterogeneity, and the software used. | 8 |
|  |  | Describe the methods used to explore possible causes of heterogeneity among study results (e.g., subgroup analysis, meta-regression). | 8 |
|  |  | Describe the sensitivity analyses that have been performed to assess the robustness of the synthesis results. | 8 |
|  | Assessment of publication bias | Describe the methods used to assess the risk of bias due to missing results in a synthesis (arising from publication biases). | 8 |
|  | Assessment of evidence confidence | Describe the methods used to assess the certainty (or confidence) in the body of evidence for each outcome. | 8 |
| **Results** | Study selection | Describe the results of the search and selection processes, from the number of records identified in the search to the number of studies included in the review, ideally using a flow chart (see figure 1). | 8-9 |
|  |  | Cite studies that appeared to meet the inclusion criteria but were excluded, and explain why they were excluded. | 8-9 |
|  | Study characteristics | Cite each included study and present its characteristics. | 9 |
|  | Risk of bias of individual studies | Present the risk of bias assessments for each of the included studies. | 9 |
|  | Results of individual studies | Present, for all outcomes and for each study: a) summary statistics for each group (if applicable), and b) the estimate of effect and its precision (e.g., credibility or confidence interval), ideally using structured tables or graphs. | 9-14 |
|  | Synthesis results | For each synthesis, briefly summarise the characteristics and risk of bias among the contributing studies. | 14 |
|  |  | Present the results of all statistical syntheses performed. If meta-analysis has been performed, present for each meta-analysis the abstract estimator and its precision (e.g., credible or confidence interval) and the measurements of statistical heterogeneity. If groups are compared, describe the direction of effect. | 14 |
|  |  | Present the results of all investigations on possible causes of heterogeneity between study results. | 14 |
|  |  | Present the results of all sensitivity analyses performed to assess the robustness of the synthesised results. | 14 |
|  | Publication bias | Submit assessments of the risk of bias due to missing results (arising from publication biases) for each synthesis assessed. | 14 |
|  | Evidence confidence | Present assessments of the certainty (or confidence) in the body of evidence for each outcome assessed. | 14 |
| **Discussion** |  | Provide an overall interpretation of the results in the context of other evidence. | 14 |
|  |  | Argue the limitations of the evidence included in the review. | 14-16 |
|  |  | Argue the limitations of the review processes used. | 14-16 |
|  |  | Argue the implications of the results for practice, policy, and future research. | 14-16 |
| **Further information** | Record and protocol | Provide the registration information for the review, including the name and registration number, or state that the review has not been registered. | 17 |
|  |  | Indicate where the protocol can be accessed, or state that no protocol has been written. | 17 |
|  |  | Describe and explain any amendments to the information provided in the registration or protocol. | 17 |
|  | Funding | Describe the sources of financial or non-financial support for the review and the role of funders or sponsors in the review. | 17 |
|  | Conflicts of interest | Declare any conflicts of interest of the review authors. | 17 |
|  | Availability of data, codes and other material | Specify which items listed below are publicly available and where they can be found: data extraction form templates, data extracted from included studies, data used for all analyses, analysis code, any other materials used in the review. | 17 |
